# Intrinsic Mechanical Cues and Their Impact on Stem Cells and Embryogenesis

**DOI:** 10.3389/fcell.2021.761871

**Published:** 2021-11-08

**Authors:** Jonna Petzold, Eileen Gentleman

**Affiliations:** Centre for Craniofacial and Regenerative Biology, King’s College London, London, United Kingdom

**Keywords:** mechanotransduction, stem cell, embryogenesis, stiffness, development

## Abstract

Although understanding how soluble cues direct cellular processes revolutionised the study of cell biology in the second half of the 20th century, over the last two decades, new insights into how mechanical cues similarly impact cell fate decisions has gained momentum. During development, extrinsic cues such as fluid flow, shear stress and compressive forces are essential for normal embryogenesis to proceed. Indeed, both adult and embryonic stem cells can respond to applied forces, but they can also detect intrinsic mechanical cues from their surrounding environment, such as the stiffness of the extracellular matrix, which impacts differentiation and morphogenesis. Cells can detect changes in their mechanical environment using cell surface receptors such as integrins and focal adhesions. Moreover, dynamic rearrangements of the cytoskeleton have been identified as a key means by which forces are transmitted from the extracellular matrix to the cell and *vice versa.* Although we have some understanding of the downstream mechanisms whereby mechanical cues are translated into changes in cell behaviour, many of the signalling pathways remain to be defined. This review discusses the importance of intrinsic mechanical cues on adult cell fate decisions, the emerging roles of cell surface mechano-sensors and the cytoskeleton in enabling cells to sense its microenvironment, and the role of intracellular signalling in translating mechanical cues into transcriptional outputs. In addition, the contribution of mechanical cues to fundamental processes during embryogenesis such as apical constriction and convergent extension is discussed. The continued development of tools to measure the biomechanical properties of soft tissues *in vivo* is likely to uncover currently underestimated contributions of these cues to adult stem cell fate decisions and embryogenesis, and may inform on regenerative strategies for tissue repair.

## Introduction

Over a century ago, tissue formation was often described in terms of mechanical cues. For example, the German surgeon Julius Wolff noted that bone adapts its inner structure in response to mechanical loading (Wolff, 1892). Later observational studies from scientists such as Eben Carey and Alfred Glücksmann concluded that the convex and concave aspects of developing bone are exposed to varying mechanical stresses, which impacted cartilage and bone formation (Carey, 1922a; Glucksmann, 1942). However, in the subsequent decades of the 20th century, much emphasis was put on understanding how highly intricate soluble biochemical cues, molecule-receptor binding interactions and their downstream transcriptional outputs control tissue formation, which together now govern much of our understanding of biology. It could be said that as a consequence, the importance of the less specific physical cues in the cellular microenvironment was somewhat overlooked.

Yet, despite these insights, it is now recognised that growth factor, chemotactic and cytokine signals alone are insufficient to explain many biological phenomena. Indeed, a cell’s mechanical landscape plays a vital role in regulating functions such as proliferation, differentiation and migration, in some cases even overriding the contribution of biochemical cues. The mechanisms whereby cells translate mechanical information from their environment into signals that alter their behaviour is termed “mechanotransduction” (Discher, 2005; DuFort et al., 2011; Walters and Gentleman, 2015). In comparison to many well characterised biochemical signals that govern cell behaviour and function, our understanding of the impact of mechanical cues on cells remains in its infancy. Despite this, the significance of tissue mechanics, and in particular matrix stiffness, in health and disease is now widely recognised (Janmey and Miller, 2011; Astudillo, 2020).

This commentary discusses the importance of intrinsic mechanical cues on adult cell fate decisions, with a focus on mesenchymal stem cells (MSC). Although externally applied extrinsic cues also play important roles in MSC differentiation, these are only briefly referred to here, but have been reviewed previously (Steward and Kelly, 2015; Vining and Mooney, 2017). Specifically, we discuss the following important questions: How do cell surface receptors such as integrins enable a cell to sense mechanical cues from its microenvironment? What is the importance of the cytoskeleton in the cellular response to mechanical cues? How do intracellular signalling pathways enable the translation of biomechanical cues into transcriptional outputs, and what is the contribution of the nucleus itself? Lastly, to put the importance of intrinsic mechanical cues into an *in vivo* biological context, a brief historical view of mechanotransduction in embryogenesis and the impact of intrinsic cues on embryonic development is outlined.

## The Role of Mechanical Cues in Driving Cell Fate

**FIGURE 1:**
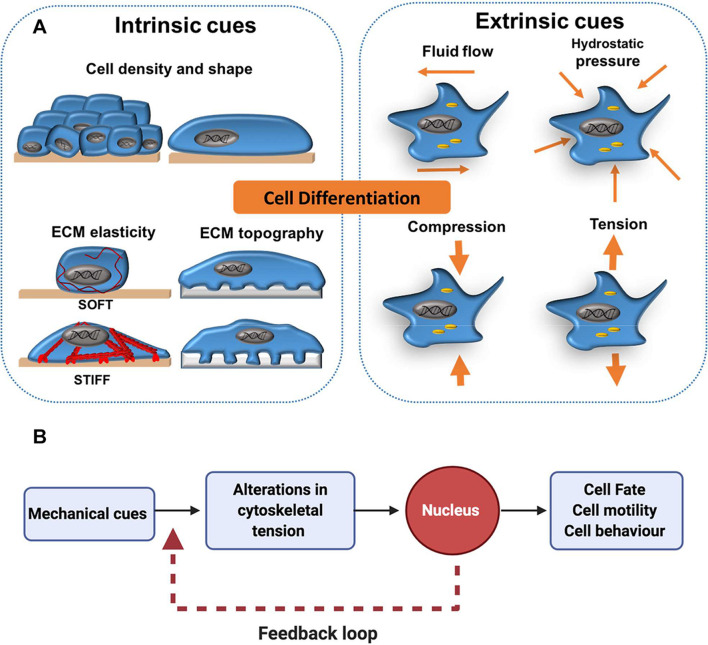
Extrinsic and intrinsic cues in mechanotransduction. **(A)** Cell differentiation has been shown to be affected by mechanical forces external to the cell (extrinsic) such as shear stress from fluid flow and more local mechanical cues (intrinsic) such as cell density, shape and elasticity of the surrounding extracellular matrix (ECM). **(B)** As a general concept, mechano-transduction involves the transfer of mechanical cues from the cell surface to the nucleus via the cytoskeleton. This activates downstream cell signalling cascades, which can influence cell fate decisions. In addition, a transcriptional feedback loop allows cells to maintain a cytoskeletal equilibrium that is responsive to changes in their mechano-environment. This is particularly important for processes like cell migration, in which continual cytoskeletal remodelling is required for persistent cell motility.

A growing body of evidence suggests that cells are able to interact with and respond to physical changes in their microenvironment (Vollrath et al., 2007; D’Angelo et al., 2011; Wisdom et al., 2018). Both extrinsic and intrinsic mechanical signals are known to regulate cell differentiation (Figure 1A). For simplicity, in this review extrinsic cues are categorised as externally applied forces that include fluid flow, compression, hydrostatic pressure and tension, whilst cell shape, density, extracellular matrix (ECM) stiffness and topography are given as examples of intrinsic cues. Importantly the mechanical landscape within organisms is highly complex and extrinsic and intrinsic cues often interrelate and cannot be decoupled from one another. Cells perceive mechanical signals in their surroundings via integrins and other cell surface molecules (Sun et al., 2016). This prompts the cellular cytoskeleton to respond by increasing or decreasing contractility to counter-balance the forces acting on the cell. Changes in cytoskeletal tension can activate downstream signalling pathways which lead to transcriptional changes that direct cell behaviour, including cell fate decisions. Direct interactions between the cytoskeleton and the nucleus also play an important role in mechanotransduction. For example, the nuclear protein lamin A accumulates in cells on stiff ECM, protecting against DNA damage (Swift and Discher, 2014; Cho et al., 2019). However, this protective effect is inhibited when the cytoskeleton is disrupted. Thus, mechanotransduction does not function as a “one-way street” and signals from the nucleus can be transferred back to the cytoskeleton to alter the way a cell perceives mechanical cues, creating a transcriptional feedback loop (Figure 1B; Swift et al., 2013; Mason et al., 2019).

Many studies in mechanobiology use bone marrow or adipose tissue-derived MSC as a model (Gomez-Salazar et al., 2020). It is important to note that despite their name, MSC do not completely fulfil the criteria of *bona fide* stem cells. Thus, MSC have also been referred to as “mesenchymal progenitor cells,” “multipotent adult stem cells” and “multipotent stromal cells” (Jiang et al., 2002; Zimmermann et al., 2003; Beltrami et al., 2007; Gomez-Salazar et al., 2020). MSC are reported to able to differentiate into several cell types such as osteoblasts, myoblasts, adipocytes and chondrocytes (Pittenger et al., 1999; McBeath et al., 2004; Deng et al., 2006; Engler et al., 2006). Their multipotency makes MSC a particularly attractive candidate for the rapidly advancing field of regenerative medicine and is the driving factor behind much of the research into this cell population.

### Cell Shape and Cell Density

The direct effect of shape on cell behaviour was observed over 40 years ago by Folkman and Moscona (1978) who developed polymer-based culture systems to alter cell shape *in vitro*. Endothelial cells cultured on thin and highly adhesive polymer layers were more spread and synthesised DNA at a faster rate compared to rounder cells cultured on thicker polymer layers (Folkman and Moscona, 1978). The development of patterned PDMS stamps, which force cells to adopt a certain morphology, identified that shape also regulates cell growth (Singhvi et al., 1994). Individual hepatocytes cultured on small adhesive islands have a round morphology, proliferate slowly and undergo apoptosis, whilst culture on larger islands promotes cell spreading and proliferation. Indeed, only 3% of hepatocytes on the smallest islands (<1,600 μm^2^) entered S phase (Singhvi et al., 1994).

More recent studies have used micropatterned substrates, in which cell shape can be tightly controlled at the micro- and nano-meter scale *in vitro* (Chen et al., 1997; Engler A. J. et al., 2004; Kumar et al., 2006). The impact of shape on cell fate was demonstrated when examining the adipogenic-osteogenic differentiation potential of MSC (McBeath et al., 2004; Engler et al., 2006; Dupont et al., 2011; Halder et al., 2012). Culture of MSC on small ECM-coated islands promotes a round cell shape and adipogenic differentiation, whilst cells spread and activate osteogenic differentiation programmes on larger islands (McBeath et al., 2004). Geometrically driven cell fate change was later confirmed by Kilian et al. (2010), who plated MSC on micropatterned surface shapes with varying cell area. Here, lipid droplets were observed in smaller, rounder cells, whilst alkaline phosphatase (ALP) expression was increased in spread cells on larger islands (Kilian et al., 2010). Importantly, the shape of these cells reflects their specialised functions *in vivo;* the round morphology of adipocytes enhances their lipid storage capabilities in adipose tissue, whereas spreading of osteoblasts maximises deposition of matrix (Parfitt, 1984; McBeath et al., 2004). In addition, cells’ aspect ratio is an important determinant of fate. Specifically, the rate of osteogenesis is ∼20% higher in MSC cultured on rectangular micro-patterns with a 4:1 aspect ratio compared to a 1:1 aspect ratio, despite the cell area remaining constant (Kilian et al., 2010). In addition, a high degree of curvature at the cell edge (flower-shaped micro-patterns) promotes adipogenesis, whilst straight cell edges (star-shaped micro-patterns) stimulates osteogenic differentiation (Kilian et al., 2010).

Cell shape and density are closely intertwined. Cells cultured at a low density have space to spread, whereas high density cultures are compact, promoting a rounded cell morphology (McBride and Knothe Tate, 2008; Wada et al., 2011). Changes in cell density directly impact on cell fate. MSC cultured at low density tend to express the osteogenic marker alkaline phosphatase (ALP), whilst high density culture promotes adipogenesis (McBeath et al., 2004). Importantly, initial plating density was found to drive lineage commitment independently of later densities; a 4-day high-density culture of MSC showed suppressed osteogenesis after re-plating at a lower density (McBeath et al., 2004). Cell density also controls morphogenesis and cell proliferation in sheets of epithelial and endothelial cells cultured *in vitro* (Nelson et al., 2005; Halder et al., 2012). Increased density at the centre of cell monolayers cultured on round FN-coated islands prevents proliferation, whilst sparsely spaced cells at island edges undergo rapid cell proliferation (Nelson et al., 2005). This difference in cell-cycle progression was attributed to a gradient of traction forces generated by cells according to their location, whereby those at the edge of the islands applied more force than cells in the centre (Nelson et al., 2005). Taken together, these studies outline the impact that cell area, aspect-ratio and density can have in determining fate.

### Effects of Extracellular Matrix Elasticity

The ECM provides both chemical and physical signals which impact on cell behaviour and fate (Eroshenko et al., 2013). Specifically, both the viscoelasticity (discussed in Section “Summary and Outlook”) and elasticity of the cellular microenvironment are known to modulate various cellular characteristics, such as shape, proliferation, differentiation and migration (Lo et al., 2000; Engler et al., 2008; Winer et al., 2008; Evans et al., 2009; Dupont et al., 2011; Kumar et al., 2017; Chaudhuri et al., 2020). “Stiffness” describes the ability of an elastic material to resist deformation when force is applied (Evans and Gentleman, 2014). In effect, this constitutes the resistance felt by a cell when it deforms its surrounding matrix (Engler et al., 2006). Stiffness is often quantified by measuring the Young’s modulus (units: pascal; Pa) of a material (Evans and Gentleman, 2014). Importantly, the Young’s modulus is a fundamental property of a material and remains the same even when the size of a material changes. Here, the terms “stiffness,” “elasticity” and “compliance” are used interchangeably to describe the same concept (Norman et al., 2021).

To better understand the effect of ECM stiffness on cell behaviour, several studies have attempted to recapitulate relevant *in vivo* stiffnesses *in vitro* by using 2D tunable polymer matrices (Engler et al., 2006; Dupont et al., 2011; Zhang et al., 2017; Zhou et al., 2017; Sun et al., 2018). A common strategy is to use polyacrylamide (PAA) hydrogels, in which varying concentrations of acrylamide and bis-acrylamide are combined to generate hydrogel matrices of varying stiffness (Pelham and Wang, 1997; Flanagan et al., 2002; Chin et al., 2020). The first well-characterised study using PAA hydrogels identified that fibroblasts and epithelial cells were less spread, irregularly shaped and lacked focal adhesions (FA) on more compliant matrices (Pelham and Wang, 1997). This finding provided early evidence that cells elicit a compliance-specific response, and provided the basis for studies that later showed stem cells to differentiate most readily on surfaces with stiffnesses that were physiologically relevant for the particular cell type (McBeath et al., 2004; Kumar et al., 2006; Venugopal et al., 2018).

The compliance of a cell’s environment modulates its morphology. In general, stiffer matrices promote cell spreading and softer matrices induce rounded cell phenotypes (Figure 1; Engler et al., 2006; McBride et al., 2008; El-Mohri et al., 2017). Cells residing in more compliant environments can easily deform their surrounding matrix and do not need to generate a large amount of force to counter-balance their matrix, thus they remain round (Knothe Tate et al., 2008). Less compliant environments resist cellular forces and are not easily deformed. Therefore, cells generate tension and respond by spreading over their matrix (McBride et al., 2008). Cell proliferation is also coupled to substrate compliance and many cells proliferate at a slower rate on softer matrices (Engler et al., 2006; Ghosh et al., 2007; Winer et al., 2008; Dupont et al., 2011; Provenzano and Keely, 2011; Wood et al., 2011). Winer et al. showed that MSC cultured on collagen I-coated PAA gels recapitulating the stiffness of bovine bone marrow (250 Pa) underwent cell cycle arrest and a reduction in DNA synthesis (Winer et al., 2008). This phenomenon has biological relevance, as this may be a mechanism by which MSC retain their stemness within the bone marrow microenvironment (Winer et al., 2008).

Stem cell differentiation and/or self-renewal has also been shown to be dependent on matrix elasticity and can be promoted on substrates with tissue-specific compliance (Engler A. et al., 2004; Georges and Janmey, 2005; Venugopal et al., 2018). For example, MSC preferentially express skeletal muscle-like myosin striations on micro-patterned substrates with a matrix compliance 8–11 kPa (Engler A. et al., 2004). This is in keeping with Atomic Force Microscopy (AFM) force spectroscopy measurements performed *ex vivo* on digitorum longus muscles in mice that identified a Young’s modulus of ∼12 kPa. Moreover, Gilbert and colleagues were able to show that the self-renewal of muscle stem cells could be enhanced on 12 kPa substrates that matched the stiffness of the native tissue (Gilbert et al., 2010). Thus, by recapitulating the mechanical compliance of the *in vivo* cellular matrix *in vitro*, a specific cellular response could be promoted.

Along similar lines, neuronal or adipogenic differentiation of MSC was found to be enhanced on softer matrices, whilst stiffer ECM promoted myocyte and osteoblast differentiation (McBeath et al., 2004; Engler et al., 2006; Dupont et al., 2011). Specifically, substrate compliances between 0.1 and 1 kPa (*in vivo* elasticity of brain tissue) promoted branched morphologies and B3 tubulin expression typical of neurons (Flanagan et al., 2002; McBeath et al., 2004), 8–17 kPa promoted striated muscle morphologies and expression of the myogenic transcription factor myogenic differentiation 1 (*MYOD1*) (Engler A. et al., 2004), and 25–40 kPa promoted osteogenic morphologies and expression of the early osteogenic transcription factor *RUNX2* (Engler et al., 2006). In fact, if MSC are pre-incubated on neurogenic matrices (0.1–1 kPa) for three weeks before switching to myogenic or osteogenic media, inductive signals from the media are over-ridden and MSC maintain a neurogenic fate (Engler et al., 2006; Halder et al., 2012). Taken together ECM elasticity and the associated cell shape changes are sufficient to drive MSC fate independently of soluble factors, although addition of induction media can further enhance this response.

**FIGURE 2:**
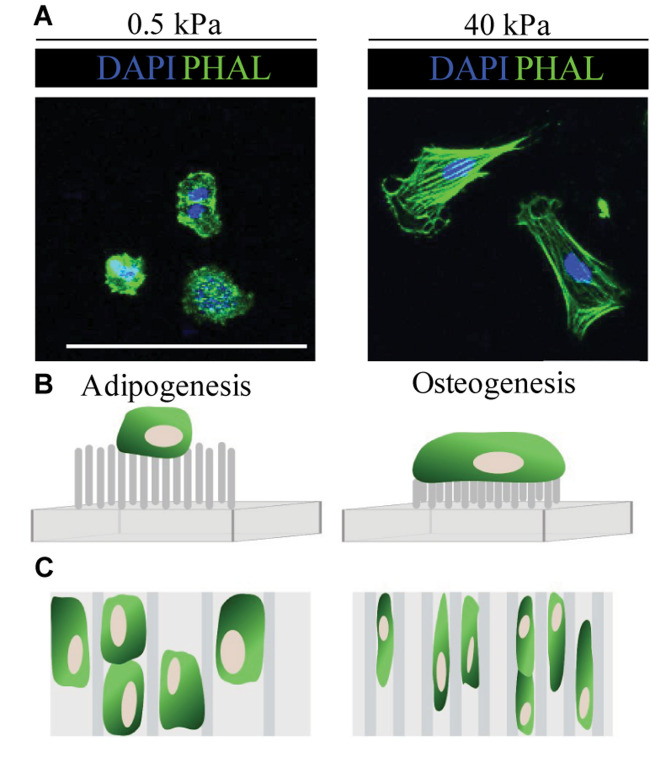
Summary of cell responses to ECM elasticity, topography and micropillars. **(A)** Fluorescence microscopy images show typical cell response on soft (0.5 kPa) and stiff (40 kPa) substrates. In general, cells (in this case embryonic neural crest cells) on soft (0.5 kPa) substrates remain rounded, whilst those on stiff (40 kPa) ECM spread and have organised F-actin fibres, as seen by the phalloidin staining (PHAL, green). **(B)** Typically, MSC cultured on long flexible micropillars respond similarly as they would on soft ECM and have a rounded morphology, whilst those on short inflexible micropillars behave as they would on stiff ECM and spread. **(C)** In general, MSC cultured on wider microgrooves show enhanced adipogenesis, whilst those on stiff substrates have an elongated morphology which promotes osteogenesis (Fu et al., 2010; Nikkhah et al., 2012; Abagnale et al., 2015). Scale bar 100 μm.

“Micropillars” of varying heights have also been developed to modulate the cell’s perceived stiffness of its substrate, whilst directly controlling the number of cell-ECM contacts *in vitro* (Figure 2; Andersson et al., 2003; Nikkhah et al., 2012; Lee et al., 2013). In general, the behaviour of cells on short pillars mirrors that on stiff PAA gels, whilst cells cultured on tall, bendable pillars behave as they would on soft ECM (Nikkhah et al., 2012). Fu et al. showed that much like on stiff surfaces, short rigid micro-posts promote MSC spreading, actin stress fibre assembly and the formation of large FA. In contrast, cells maintain a rounded phenotype and disorganised actin structure on longer micro-posts (Fu et al., 2010). Here, micropillar-induced specification did not occur in normal differentiation medium, but when supplemented with adipo-osteogenic differentiation factors, rigid pillars promoted osteogenic and soft pillars enhanced adipogenic differentiation.

It is important to note that cellular responses to ECM stiffness are not universal. Although for many cell types, differentiation is enhanced on tissue-specific ECM stiffnesses, this is not always the case. For instance, it has been reported that the expansion of undifferentiated embryonic fibroblasts occurs independently of substrate stiffness (Ali et al., 2015). In addition, human blood cells such as neutrophils appear to be insensitive to the compliance of their environment and spread equally on a range of matrix stiffnesses from 180 Pa to 2.8 kPa (Discher, 2005; Yeung et al., 2005). Lastly, the differentiation state of cells may play a role in how responsive they are to mechanical cues (Eroshenko et al., 2013). Although mature fibroblasts and endothelial cells alter their shape when exposed to different ECM stiffnesses, this is not the case in undifferentiated ESC. Here, no change in undifferentiated ESC shape was observed within 12 h of culture on matrices with varying compliance (Eroshenko et al., 2013). Taken together, cell responses to ECM elasticity appear to be fundamentally different depending on the cell type, so conclusions one cannot necessarily be extrapolated to other cell populations.

### Extracellular Matrix Topography

In addition to ECM stiffness and geometry, topographic changes to the cellular environment impact on cell behaviour and fate (Chen et al., 2012; Eroshenko et al., 2013; Abagnale et al., 2017, 2015). Thus far, we have discussed cellular responses to the mechanical properties of flat 2D surfaces; however, during embryogenesis and adult homeostasis, cells are likely to encounter a varying topographic landscape (Abagnale et al., 2017; Murakami et al., 2017). Micro-and nano-printing techniques using microgrooves, ridges and thin polymer fibres, have helped to delineate the impact of surface topography on cell differentiation (Chen et al., 2012; Chen and Zhu, 2013). In general, the presence of grooves and ridges increases cell attachment, proliferation and alignment in comparison with flat controls (Peerani et al., 2007; Yim et al., 2007; Chen et al., 2012; Goetzke et al., 2018). For example, using microgrooved polyimide substrates, Abagnale et al. report that MSC cultured on wider grooves (15 μm) undergo adipogenesis, whilst those on thinner grooves (2 μm) differentiate into osteoblasts (Figure 2; Abagnale et al., 2015). Notably, altering ridge width was not sufficient to induce terminal differentiation in MSC *per se*, but directed differentiation toward a particular lineage. In this case, soluble growth factors were required to fully induce adipogenic or osteogenic fate (Abagnale et al., 2015).

Notably, defined and straight microgrooves are unlikely to replicate the complexity of the *in vivo* environment, a problem partly overcome by the development of nanorough surfaces. Here, reactive ion etching is used to generate nanorough surfaces, with a surface roughness between 1 and 150 nm (Chen et al., 2012). In one study, 7-day culture on nanorough surfaces stimulated ESC differentiation, as indicated by a reduction in the number of Oct3/4 positive cells from 93% on smooth glass to 37% on 150 nm nanorough glass (Chen et al., 2012). Not only is cell morphology and differentiation sensitive to surface topography, the release of cytokines to fight bacterial infection is also affected. Epithelial cells seeded onto microgrooves or nanopillars released fewer proinflammatory cytokines and chemokines in comparison to flat controls, despite identical surface chemistry between conditions (Andersson et al., 2003). This highlights the far-reaching impact of the topographical environment. As with cell shape and ECM stiffness, cell responses to topological cues appears to be cell-type dependent, thus conclusions from one cell type cannot be extrapolated to another.

## Mechanisms of Mechanotransduction – Mechanosensors

**FIGURE 3:**
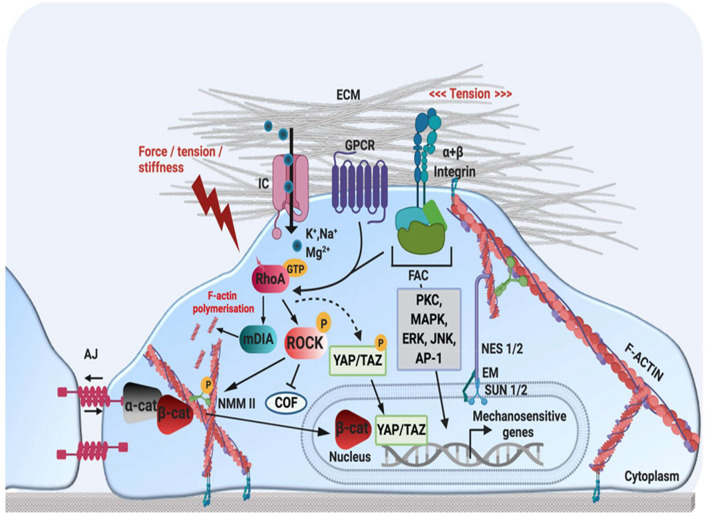
Schematic representation of mechanotransduction pathways. Mechanical stimuli are perceived by mechano-sensors at the cellular-ECM interface, such as integrin-FA complexes, GPCR, AJ and stretch-activated ion channels. This activates several cellular signalling pathways involving kinases or transcription factors (MAPK, ERK, JNK, PKC, AP-1), as well as Rho small GTPases (RhoA). RhoA-GTP regulates actin structure by (1) activating mDia to promote actin polymerisation (2) activating ROCK, which enhances actin contractility by activating NMM II phosphorylation, and (3) preventing actin disassembly by inhibiting the actin-severing protein COF. The remodelling of F-actin and increased cytoskeletal tension also regulates YAP/TAZ, which translocate to the nucleus in response to mechanical strain. At AJ, cadherin-actomyosin connections form via α-cat and ß-cat. An increase in tension at cell-cell contacts induces unfolding of α-cat, which promotes recruitment of AJ-stabilisation proteins such as vinculin. In response to a loss of cell-cell adhesion or mechanical stimulation, ß-cat can translocate to the nucleus, to activate mechanosensitive genes. Nuclear mechano-transduction occurs via the LINC complex, which directly couples the nuclear envelope to the cytoskeleton. NES 1/2 form a link between actin and SUN 1/2 proteins in the perinuclear space, which interact with the nuclear lamina via EM and lamin A. Nesprin proteins also connect the nuclear lamina with intermediate filaments and microtubules (not depicted here). JNK, c-Jun N-terminal kinase; PKC, protein kinase C; AP-1, activator protein 1; FAC, focal adhesion complex; GCPR, G-protein coupled receptor; IC, ion channel; ECM, extracellular matrix; AJ, adherens junction; α-cat, alpha-catenin; ß-cat, beta-catenin; YAP, yes associated protein; TAZ, WW domain-containing transcription regulator protein 1 NES 1/2, nesprin-1/2; SUN 1/2, sun-domain containing protein 1/2; EM, emerin; AP-1, activator protein 1; ERK, extracellular-receptor kinase; ROCK, rho-associated protein kinase; RhoA, ras homolog family member A; COF, cofillin; NMM II, non-muscle myosin II. Created using BioRender.com.

Section 3 described the behavioural responses of cells to intrinsic mechanical cues. However, the intracellular mechanisms by which mechanical cues are translated into transcriptional outputs are less well understood. In general, mechanical signals are initially perceived by membrane-embedded proteins acting as “stiffness-sensors” such as integrins, FA, G-protein coupled receptors (GPCR), cadherins and ion channels (Aragona et al., 2013; Dasgupta and McCollum, 2019). This activates Rho-ROCK, FAK and integrin-mediated signalling pathways. Subsequently, the cytoskeleton responds by changing its structure to increase or decrease cellular contractility. Ultimately, these cytoskeletal changes activate downstream signalling pathways, such as YAP/TAZ and MRTF-SRF signalling, leading to changes in cell behaviour and fate. Figure 3 provides an overview of some of the most important mechano-transduction pathways identified to date.

### Integrin and Focal Adhesion Signalling

Integrins are transmembrane receptors that consist of non-covalently bonded α and ß subunits at the cell membrane, which directly tether the cytoskeleton to the ECM. Many changes to the mechanical properties of the ECM will be perceived by these transmembrane receptors. For instance, if the rigidity of the ECM is increased, this is felt via integrin receptors on the cell surface. These integrin receptors are associated with the actin cytoskeleton via the “integrin adhesome,” which consists of several proteins (Winograd-Katz et al., 2014). Thus, the integrin adhesome enables changes in the cellular microenvironment to be transmitted to the cytoskeleton and *vice versa* (Ingber, 2006; Kumar et al., 2006). In stiff microenvironments, the cell responds by re-arranging its actin cytoskeleton and strengthening its stress fibres to balance out the forces exerted by the ECM (Ingber, 2006). This maintains a tensional equilibrium between the cell and its surrounding microenvironment, whereby stiffness-induced changes in cytoskeletal tension are transmitted back to the ECM via FA and integrin receptors, enabling cells to remodel their surrounding matrix (Ingber, 2006).

**FIGURE 4:**
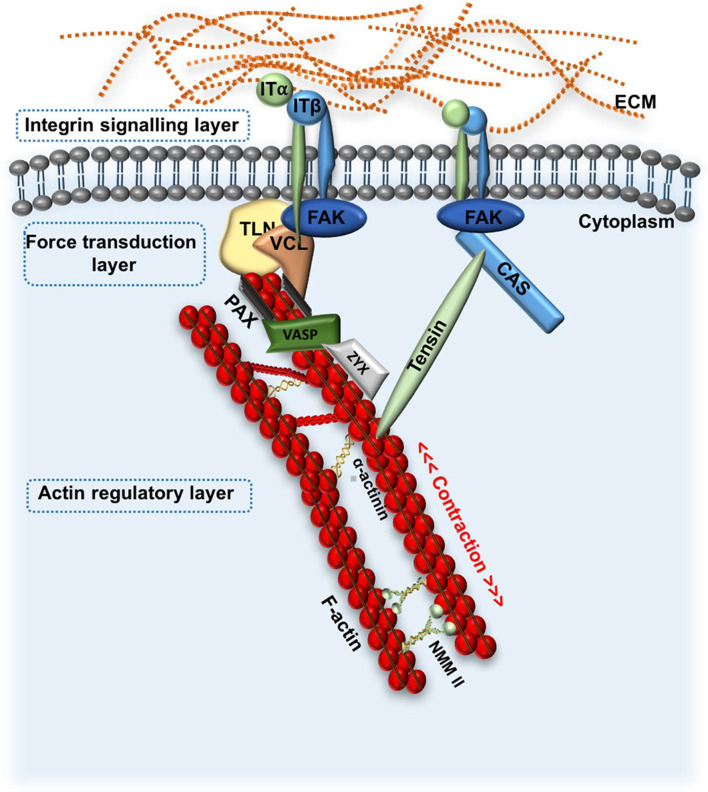
Schematic representation of focal adhesion kinase signalling. FA signalling: FAK is recruited to integrin clusters at the cell-ECM boundary in response to changes to ECM stiffness, or other physical cues. This initiates formation of the FA complex by recruitment of various proteins such as TLN and VCL and CAS, which transduce mechanical stimuli from the ECM to the cellular cytoskeleton. VASP, Zyx and α-actinin directly regulate actin assembly. Three general FA layers are depicted, including the integrin signalling layer, force transduction layer and actin regulatory layer. FA, focal adhesion; ECM, extracellular matrix; ITα;ITß, integrin subunit α and ß; FAK, focal adhesion kinase; TLN, talin; VCL, vinculin; Zyz, zyxin; NMM II, non-muscle myosin II; VASP, vasodilator-stimulated phosphoprotein.

FA are the main site of interaction between ECM-bound integrins and the actin cytoskeleton, providing a form of molecular bridge between the ECM and the cell (Matthews et al., 2004; Martino et al., 2018). This enables integrins and FA to mediate several processes such as cell adhesion, migration, cell-ECM force transmission, cytoskeletal re-arrangements and signal transduction (Kanchanawong et al., 2010; Bays and DeMali, 2017; Martino et al., 2018). To date, over 50 proteins have been associated with FA sites; some of the most well-characterised components of FA complexes include the non-receptor tyrosine kinase focal adhesion kinase (FAK), the adaptor proteins paxillin, talin, vinculin, zyxin, vasodilator–stimulated phosphoprotein (VASP) and the microfilament protein α-actinin (Bays and DeMali, 2017; Burridge, 2017). Using 3D super-resolution fluorescence microscopy, Kanchanawong et al. identified three vertical FA layers; the uppermost “integrin signalling layer,” the central “force transduction layer,” and the innermost “actin regulatory layer,” each composed of different interacting proteins (Figure 4; Kanchanawong et al., 2010). This “integrin adhesome” spans 20 nm across the plasma membrane and provides a “snapshot” view of the position of FA proteins (Kanchanawong et al., 2010). Later evidence shows that when activated, some proteins such as vinculin and zyxin are mobilised from their position in one layer (in this case the signalling layer) to another (in this case actin regulatory layer). This active redistribution of proteins helps to propagate mechanical signals from the ECM to the cytoskeleton (Yoshigi et al., 2005; Case et al., 2015; Sun et al., 2016).

FA proteins are highly sensitive to the cellular microenvironment, and are readily recruited and assembled at the integrin binding site in response to an increase in ECM stiffness (Smith et al., 2013). This stabilises the strain site and reinforces the cytoskeleton (Smith et al., 2013). Seong et al. visualised FAK activity using a FAK-FRET biosensor in various tumour cell lines cultured on surfaces with varying stiffnesses and concluded that FAK activity is directly proportional to increasing substrate stiffness (Seong et al., 2013). This was correlated with increased cell traction, confirming that cells in stiffer environments exert a higher traction to interact with their less compliant surroundings (Seong et al., 2013). The recruitment of the adaptor protein paxillin to FA sites is also known to be tension sensitive; a lack of tension reduces paxillin at FA and prevents actin polymerisation, leading to a lack of stress fibre repair and stress fibre breaks (Smith et al., 2013; Martino et al., 2018). Thus, effective recruitment of FA proteins is essential for the reinforcement of the cytoskeleton in response to mechanical cues.

In addition to recruitment of FA adaptor proteins, application of force can induce conformational changes to promote their interaction. Indeed, vinculin forms a link between talin and actin, which is essential for cells to strengthen their FA and generate traction forces (Atherton et al., 2015; Martino et al., 2018). The adhesion protein talin has several vinculin binding sites, but these remain unavailable to vinculin in the absence of force (Rahikainen et al., 2017). However, by computationally inducing changes to the stability of talin, Rahikainen et al. showed that mechanical forces are transmitted through talin as the FA site matures, which promotes unfolding of the protein. As a result, binding sites for vinculin are made available and subsequent vinculin accumulation strengthens the adhesion complex (Rahikainen et al., 2017).

FA and their associated adaptor proteins play an important role in creating the ECM–cytoskeleton–nuclear signalling axis. As mentioned, several FA proteins, such as zyxin, VASP and vinculin can be redistributed to actin stress fibres within the cell when mechanical force is applied (Yoshigi et al., 2005; Case et al., 2015). Some reports show that zyxin and paxillin can detach from FA sites and translocate directly to the nucleus to initiate specific gene expression changes (Cattaruzza et al., 2004; Zhou et al., 2017). For instance, in vascular smooth muscle cells, zyxin dissociates and shuttles to the nucleus when cyclic stretch is applied to cells *in vitro*, modulating mechano-responsive genes such as those for endothelin B and tenascin-C (Cattaruzza et al., 2004). In addition, nuclear transport of paxillin is known to promote DNA synthesis and cell proliferation in cervical cancer cells (Dong et al., 2009). Taken together, these studies demonstrate the important role integrins and FA play in the transmission of mechanical cues and their translation into biochemical responses.

### The Cytoskeleton

The cytoskeleton is a dynamic structure composed of F-actin stress fibres, microtubules and intermediate filaments, which control cell movement, shape and homeostasis (Fletcher et al., 2010; Hoffman et al., 2011; Martino et al., 2018). Contraction of the cytoskeleton is mediated by F-actin fibres and NMM II contractile units, which form direct links with integrins and FA at the cell membrane to transmit forces from the ECM to the cell and *vice versa* (Engler et al., 2006; Naumanen et al., 2008; Kilian et al., 2010). The cytoskeleton exerts tension in a similar way to which muscles contract; as NMM II contracts, actomyosin filaments slide over one-another and contract (Steward and Kelly, 2015). As such, the cytoskeleton can “feel” and counterbalance extracellular forces applied to the cell by generating intracellular tension. Subsequently, this increases or decreases the traction forces applied by the cell to its surrounding matrix, a phenomenon described as “mechano-sensing” (Evans and Gentleman, 2014).

Changes in ECM stiffness have a striking effect on F-actin structure and assembly. Cells on stiff ECM cannot deform their matrix and generate highly organised linear arrays of F-actin fibres, whilst cells on soft ECM deform their surrounding matrix and do not exhibit pronounced cytoskeletal F-actin fibres (Georges and Janmey, 2005; Engler et al., 2006; Ghosh et al., 2007; Seong et al., 2013; Evans and Gentleman, 2014). For instance, human dermal fibroblasts cultured on stiff PAA gels (∼5 kPa) have highly organised F-actin fibres, whilst F-actin filaments are irregular in cells cultured on soft matrices (550 Pa) (Ghosh et al., 2007). This study also characterised the traction forces generated by fibroblasts on their ECM by dissociating fibroblasts from PAA hydrogels and measuring the subsequent displacement of 40 nm fluorescent beads embedded within the substrates. The traction forces exerted by the fibroblasts on their matrix as well as the stiffness of the cells themselves increased as the matrix became less compliant (Ghosh et al., 2007). Changes to ECM topography also impact on F-actin assembly (Halder et al., 2012). This is particularly noticeable in cells cultured on micro-patterned linear grooves where F-actin fibres arrange themselves parallel to the grooves (Engler A. et al., 2004; Halder et al., 2012). Notably, F-actin stress fibre size, strength, and curvature are directly linked to the number and spatial distribution of cell-ECM adhesion sites (Théry et al., 2006). This matrix-specific cytoskeletal response allows cells to appropriately interact with and deform their surrounding matrix.

Small molecule cytoskeletal inhibitors have helped to elucidate the role of the cytoskeleton in propagating mechanical signals *in vitro*. Common inhibitors include blebbistatin (inhibits NMM II), Y-27632 (inhibits Rho kinase; ROCK) and Latrunculin A (inhibits actin polymerisation). These inhibitors have helped identify the role of cytoskeletal tension in cell lineage specification (Engler A. et al., 2004; Engler et al., 2006; Kumar et al., 2006). For instance, treatment with blebbistatin prevents the stiffness-induced differentiation of MSC, which demonstrates the integral role of the cytoskeleton in mediating the mechano-sensory response of MSC (Engler et al., 2006). As mentioned previously, MSC cultured on flower- or star-shaped patterns promote adipogenic (72%) or osteogenic (67%) cell fates, respectively (Kilian et al., 2010). However, when cytoskeletal tension is inhibited, adipogenic differentiation is favoured on both shapes (Kilian et al., 2010). In contrast, osteogenesis is promoted independently of cell shape when actomyosin contractility is pharmacologically enhanced (Kilian et al., 2010). Cytoskeletal inhibitors have also proven fundamental in determining the longevity of tension-mediated cell fate changes (Fu et al., 2010). Indeed, a 12-h Y27632-treatment of MSC on ridged micro-pillars suppressed osteogenic differentiation for up to 7-days post-treatment (Fu et al., 2010). The studies discussed here indicate that the cellular cytoskeleton, traction forces and cell stiffness act in a feedback loop and respond to changes in substrate dynamics to create an equilibrium between cell and matrix tension (Ghosh et al., 2007).

### Rho/Rho-Associated Protein Kinase/Non-muscle Myosin II Signalling

Rho/ROCK signalling is one of the main pathways mediating the cytoskeletal responses described above. This Rho family of GTPases (RhoA, Rac and Cdc42) is responsible for the organisation of actin cytoskeletal stress fibres and the formation of lamellipodia and filopodia (Nobes and Hall, 1999; Amano et al., 2010). Rho and ROCK can directly associate with actin stress fibres and when Rho is active (Rho-GTP), it signals via ROCK to increase cytoskeletal contraction in response to force (Leung et al., 1996; Amano et al., 1997; Katoh et al., 2011). When ROCK is active, stress fibres and FA are well-defined whilst ROCK inhibition disrupts F-actin stress fibres and reduces contractile tension after just 1 h (Katoh et al., 2001).

ROCK induces and maintains stress fibre contraction via various mechanisms (Amano et al., 2010; Katoh et al., 2011, 2001; Julian and Olson, 2014). For instance, ROCK phosphorylates myosin II light chain (MLC) and activates myosin ATPase, which mediates the interaction between MLC and F-actin to induce actomyosin contractility (Julian and Olson, 2014). Furthermore, ROCK inactivates myosin phosphatase, which prevents this kinase from dephosphorylating MLC, maintaining the activity of MLC (Julian and Olson, 2014). ROCK kinases also phosphorylate LIM kinases and subsequently inactivate cofilin, preventing this protein from depolymerising actin filaments (Sumi et al., 2001). Thus, inhibition of cofilin results in an overall increase in the number of cellular actin filaments and cytoskeletal tension (Sumi et al., 2001). In summary, Rho, ROCK and MLC work together to modulate force-induced actomyosin contraction.

Rho/ROCK signalling has been implicated in cell fate decisions in multiple cell types (Sordella et al., 2003; McBeath et al., 2004; Woods et al., 2005). Indeed, the fundamental role of ROCK signalling in MSC differentiation in response to cell shape was identified in 2004. Here, transfection of MSC with active ROCK was sufficient to induce osteogenic fate independently of cell shape (McBeath et al., 2004). In addition, pharmacological inhibition of ROCK prevented stress fibre formation and osteogenesis. Interestingly, the authors conclude that both cell shape and RhoA signalling are necessary, but that neither is sufficient to drive cell fate in MSC (McBeath et al., 2004). Later studies in human fibroblasts confirmed that high ROCK activity is associated with stiff ECM and osteogenesis, whilst soft matrix is associated with low ROCK activity and adipogenic fate (Katoh et al., 2011). Rho/ROCK signalling also plays an important role in chondrogenesis. Inhibition of RhoA or ROCK increases glycosaminoglycan production and mRNA expression of the chondrogenic marker *SOX9* in MSC cultured *in vitro* (Woods et al., 2005). Moreover, ROCK inhibition in the chondrogenic cell line ATDC5 promotes a round cell morphology and an increase in cortical actin, which are typical hallmarks of the chondrogenic phenotypes (Woods et al., 2005). In a later study, the hypoxia-mediated enrichment of chondrogenic markers on soft PAA gels was prevented by inhibition of ROCK (Foyt et al., 2019). This suggests that Rho/ROCK signalling may underpin the effects of hypoxia in this context.

Rho signalling has also been implicated in the switch between adipogenic and myogenic differentiation programmes in MSC (Sordella et al., 2003; McBeath et al., 2004). Several findings support the notion that Rho activity promotes myogenesis, whilst RhoA inhibition induces adipogenesis. MSC cultured in ROCK inhibitor promoted adipogenesis (Sordella et al., 2003); however, expression of a constitutionally active Rho GTPase (RhoV14), which acts upstream of ROCK, reduces adipogenesis. This effect is mediated by the insulin growth factor (IGF) pathway, whereby IGF-1 promotes Rho activation which drives myogenesis. Mechanical cues such as oscillatory fluid flow have been shown to directly activate RhoA and downstream ROCK in murine MSC, and induce the expression of the osteogenic marker *Runx2*. Inhibition of RhoA and ROCK independently of one another, found that both are required for flow-induced *Runx2* expression (Arnsdorf et al., 2009). In conclusion, these studies illustrate the integral role that RhoA/ROCK signalling plays in the transmission of mechanical cues to drive cell differentiation.

### Yes-Associated Protein/TAZ Signalling

**FIGURE 5:**
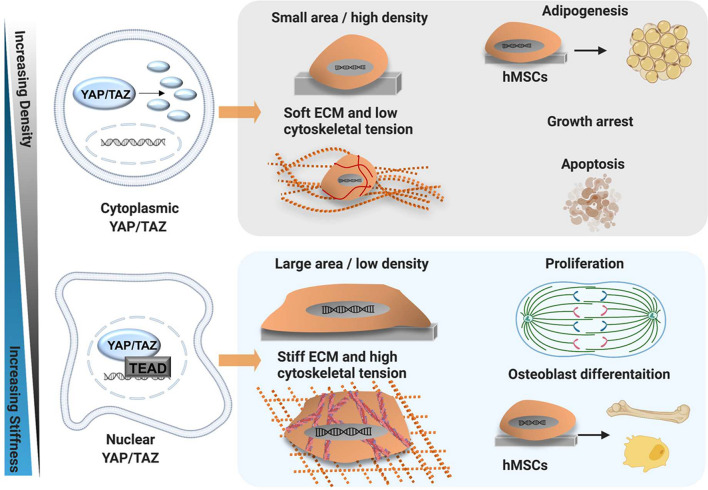
YAP/TAZ mechanism of action. Schematic showing mechanical regulation of YAP/TAZ activity and modulation of cell behaviour by YAP/TAZ in MSC. Osteogenesis and skeletal muscle fates are promoted by stiff ECM and a low cell density, allowing MSC to spread and generate cytoskeletal tension via F-actin stress fibres. The stiff matrix promotes stress fibre formation and YAP/TAZ nuclear translocation. Conversely, adipogenic fates are promoted by soft ECM and high cell-cell contact. The soft matrix prevents stress fibre formation, thus MSC cannot generate tension and display only cortical actin. As such, YAP/TAZ are retained in the cytoplasm, undergo proteasomal degradation and are rendered inactive, promoting adipogenesis. Created using Biorender.com.

**FIGURE 6:**
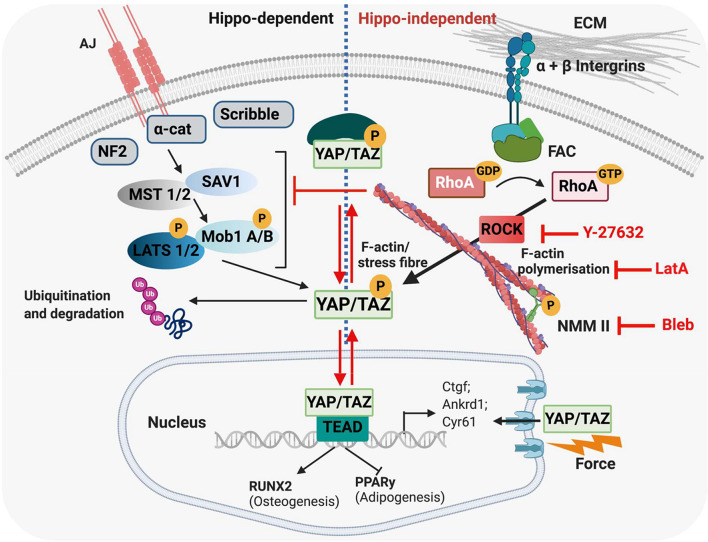
Hippo-dependent and Hippo-independent regulation of YAP/TAZ. YAP/TAZ are known to be regulated via the HIPPO signalling pathway and by a mechanically regulated HIPPO-independent mechanism. **(Left)** HIPPO control of YAP and TAZ. The HIPPO pathway regulates organ growth as well as cell proliferation, migration and differentiation. In tightly packed tissues, proliferation is regulated by contact inhibition via the HIPPO pathway. Tight junctions and adherens junctions between cells interact with and activate MST1/2, which recruit SAV1, and subsequently phosphorylate LATS1/2. This phosphorylation is facilitated by the scaffold proteins MOB1 A/B and NF2. In turn, LATS1/2 phosphorylate YAP/TAZ, leading to cytoplasmic sequestering of these proteins, and their eventual ubiquitination/degradation. F-actin has been proposed to regulate YAP/TAZ localisation via the HIPPO pathway by inhibiting LATS1/2 and/or upstream factors, thus preventing phosphorylation and cytoplasmic retention of YAP/TAZ. **(Right)** ECM stiffness also regulates YAP/TAZ in a HIPPO-independent mechanism. Cells interact with their ECM via integrins; in stiff environments, focal adhesion assembly is promoted, which activates Rho-ROCK signalling, which in turn activates F-actin stress fibre formation and translocation of YAP/TAZ to the nucleus, where these proteins regulate gene expression via activation of TEAD1-4. External application of force to the nucleus has also been shown to open nuclear pores and allow increased YAP/TAZ entry into the nucleus. Cytoskeletal inhibitors affect different parts of the mechanotransduction pathway; Y-27632 inhibits ROCK, Latrunculin A inhibits F-actin polymerisation and Blebbistatin inhibits Myosin (all depicted in red). MST1/2, mammalian ste-20-like kinases 1/2; SAV1, salvador family WW domain containing protein 1; LATS1/2, large tumour suppressors 1/2; MOB1 A/B, MOB kinase activator 1A; NF2, neurofibromatosis type 2; TEAD1-4, TEA domain family member 1-4; FAC, focal adhesion kinase. Created using Biorender.com.

The protein homologues yes-associated protein (YAP) and WW domain-containing transcription regulator protein 1 (TAZ) are key components of the HIPPO signalling cascade, which regulates organ size, cell proliferation, differentiation and migration in several systems (Dupont, 2016; Hindley et al., 2016; Manning et al., 2020). When Hippo signalling is active, YAP and TAZ are phosphorylated by large tumour suppressor kinase 1/2 (LATS1/2), which induces YAP/TAZ ubiquitination and degradation and/or sequesters the proteins to the cytoplasm. When the HIPPO signalling is inactive, YAP and TAZ are not phosphorylated and translocate to the nucleus, where they bind to TEAD regulatory elements and activate transcriptional programmes to promote cell growth and proliferation (Figure 5). In principle, nuclear YAP/TAZ promotes proliferation whilst contact inhibition induces cytoplasmic and transcriptionally inactive YAP/TAZ, reducing proliferation (Piccolo et al., 2014). In reality, nuclear-cytoplasmic shuttling of YAP/TAZ is more complex and occurs via multiple regulatory pathways. For instance, these proteins can be phosphorylated by other kinases, for example protein kinase B (AKT) and c-Jun N-terminal kinases (JNK) and are regulated by the ß-catenin degradation complex during WNT signalling (Basu et al., 2003; Azzolin et al., 2014; Piccolo et al., 2014). In addition, YAP/TAZ activity has been found to be regulated via HIPPO-dependent and HIPPO-independent mechanisms (Figure 6; Zhao et al., 2008; Dupont et al., 2011; Aragona et al., 2013; Dobrokhotov et al., 2018).

In 2011, pioneering work by Dupont et al. categorised YAP and TAZ as “mechano-sensors.” They identified that in MSC, the localisation of YAP/TAZ changes in response to mechanical cues such as shape, density, ECM stiffness and cytoskeletal tension (Dupont et al., 2011). Specifically, small micro-patterned islands, low ECM stiffness, high cell density and a rounded shape promoted cytoplasmic retention of YAP/TAZ, while larger islands, a high matrix stiffness, sparse cell density and spreading promoted nuclear accumulation of YAP/TAZ (Dupont et al., 2011). Moreover, YAP/TAZ localisation impacted MSC fate. Nuclear YAP was found to promote osteogenesis, while cytoplasmic localisation drove adipogenesis (Dupont et al., 2011; Dupont, 2016). Importantly, overexpression of YAP/TAZ *in vitro* promotes osteogenic differentiation and cell proliferation in cells on soft ECM, and is thus sufficient to “trick” cells into behaving as they would on a stiff matrix (Dupont et al., 2011). In short, YAP/TAZ sense mechanical cues and also mediate the cellular response to mechanical stimulation, a mechanism which is conserved across multiple cell types (Engler et al., 2006; Dupont et al., 2011; Wada et al., 2011; Halder et al., 2012; Aragona et al., 2013; Galarza Torre et al., 2018; Meng et al., 2018).

Wada et al. (2011) reported that actomyosin tension regulates YAP/TAZ through LATS1/2-dependent phosphorylation of YAP. It was proposed that signals from F-actin stress fibres either directly inhibit or function upstream of LATS 1/2, which prevents YAP/TAZ phosphorylation (Wada et al., 2011). In endothelial cells, pharmacological disruption of F-actin led to a decrease in nuclear YAP localisation. However, when endothelial cells were transfected with a kinase-defective form of LATS2 and F-actin was inhibited, nuclear YAP was maintained. The authors conclude that stress fibres regulate YAP via HIPPO signalling, although the possibility that F-actin also functions independently of HIPPO could not be excluded. Stiff ECM also activates a FAK signalling pathway (β1-integrin–FAK–Src–PI3K–PDK1), which directly inhibits LATS1/2 activity and promotes nuclear YAP localisation (Dobrokhotov et al., 2018; Lachowski et al., 2018).

However, YAP/TAZ have also been found to be modulated via cytoskeletal tension, cell shape, density and ECM stiffness *independently* of HIPPO signalling pathways (Aragona et al., 2013; Dobrokhotov et al., 2018). This phenomenon has been reported in MSC, mouse embryonic fibroblasts, keratinocytes and mammary epithelial cells (Dupont et al., 2011; Halder et al., 2012). Disruption of actomyosin tension inactivates YAP/TAZ and promotes their cytoplasmic localisation, which suggests these proteins are directly regulated by the cytoskeleton (Dupont et al., 2011; Wada et al., 2011; Zhao et al., 2012). YAP/TAZ expression can be modulated by F-actin capping and severing proteins, which prevent actin polymerisation; siRNA-mediated knock-out of the actin-capping proteins reactivated mRNA expression of the YAP/TAZ target genes on soft matrices and in dense cultures (Aragona et al., 2013). In addition, LATS1/2 knockdown did not restore nuclear YAP/TAZ activity in cells treated with a cytoskeletal inhibitor, or cells cultured on soft ECM (Dupont et al., 2011; Aragona et al., 2013). This suggests that mechanical control of YAP/TAZ activity is predominantly regulated by cytoskeletal signals, which may dominate over HIPPO-dependent signalling. Moreover, physical cues and F-actin structure can also alter the responsiveness of YAP/TAZ to inputs from WNT or GPCR signalling (Aragona et al., 2013). Ultimately, this implies that cells require an appropriate cytoskeletal structure to control YAP/TAZ transcriptional activity; however, the exact mechanisms are not yet well characterised (Piccolo et al., 2014).

Additional regulators of YAP/TAZ activity such as calveolin-1 (CAV1), as well as the nucleus itself have recently been described (Elosegui-Artola et al., 2017; Meng et al., 2018; Moreno-Vicente et al., 2018). CAV1 controls YAP via a HIPPO-independent mechanism; mouse embryonic fibroblasts deficient of CAV1 exhibit a disorganised actin cytoskeleton, cytoplasmic YAP and reduced expression of YAP targets (Moreno-Vicente et al., 2018). CAV1 was also found to directly control the response of YAP/TAZ to cytoskeletal tension via direct interaction with YAP (Moreno-Vicente et al., 2018). In addition, stiff ECM has been found to drive YAP/TAZ into the nucleus by opening nuclear pores which can occur independently of cytoskeletal contraction (see Section “Nuclear Mechanotransduction”; Elosegui-Artola et al., 2017; Dobrokhotov et al., 2018).

### Serum Response Factor Signalling and Myocardin-Related Transcription Factors

Myocardin-related transcription factors (MRTF) and serum response factor (SRF) also play important roles in modulating gene expression in response to biophysical cues (Olson and Nordheim, 2010; Costa et al., 2012). MRTFs bind to nuclear SRF, which activates downstream SRF-responsive genes, many of which are involved in the regulation of the cellular actomyosin structure. MRTFs are normally sequestered to the cytoplasm when bound to G-actin in the presence of low actin polymerisation (Sotiropoulos et al., 1999; Posern et al., 2002). However, when cells are mechanically stimulated, MRTF is released from G-actin and translocates to the nucleus, where it directly interacts with SRF (Sotiropoulos et al., 1999; Miano et al., 2007; Olson and Nordheim, 2010). The downstream targets of SRF are not limited to cytoskeletal genes. SRF also regulates smooth muscle differentiation by binding to the CArg box element of myocardin (MYOCD) (Miano et al., 2007). Several stretch-sensitive signalling pathways, such as the ERK1/2 pathway have been implicated in smooth muscle differentiation. ERK1/2 mediates its responses by phosphorylating ternary complex factors, which bind to SRF and activate early smooth muscle gene-expression (Ball and Price, 1995; Hellstrand and Albinsson, 2005).

### Nuclear Mechanotransduction

The “LINC complex” (linker of nucleoskeleton and cytoskeleton) directly links cytoskeletal components to the nuclear surface and has received much attention in recent years for its role in the direct transmission of ECM force to the nucleus (Crisp et al., 2006; Neelam et al., 2015; Martino et al., 2018). This complex contains several proteins including sun-domain containing protein 1/2 (SUN 1/2), nesprin and lamins, which directly anchor cytoskeletal elements such as microtubules, intermediate filaments and F-actin to the nuclear envelope (Crisp et al., 2006; Wang et al., 2009; Swift et al., 2013; Neelam et al., 2015). Mechanosensitive proteins, such as nuclear lamins, are responsible for driving many cellular responses to stiffness by either changing their confirmation, undergoing post-translational modifications or altering their subcellular localisation (Buxboim et al., 2014; Cho et al., 2019). For instance, increases in matrix stiffness and subsequent changes to myosin II activity lead to increased dephosphorylation of the nucleoskeletal protein lamin A, which regulates its turnover and the properties of the nuclear envelope (Buxboim et al., 2014).

Nuclear shape is also important in mechanotransduction. For example, micropipette-induced deformation of nuclear shape can be enhanced when cells are treated with inhibitors of intermediate filaments (Neelam et al., 2015). This suggests that intermediate filaments aid in the control of nuclear deformation (Neelam et al., 2015). Changes to cytoskeletal structure are known to directly impact on nuclear membrane shape, ion channels and the structure of nuclear pores, which in turn affects gene expression (Feldherr and Akin, 1990; Wang et al., 2009). However, the mechanisms through which mechanical cues are translated to the nucleus via the cytoskeleton are not yet well understood. A recent report proposes that force transmission to the nucleus occurs independently of the cytoskeleton. Indeed, cells cultured on stiff ECM had flatter and stretched nuclei, which in turn stretched nuclear pores and increased nuclear YAP import (Elosegui-Artola et al., 2017). Interestingly, force application to the nucleus via AFM was sufficient to translocate YAP to the nucleus independently of FA and when components of the cytoskeleton were inhibited (Elosegui-Artola et al., 2017). Thus, direct force transmission from the ECM to the nucleus is a novel and alternative mechanism for controlling gene expression.

## The Role of Intrinsic Mechanical Forces in Embryonic Development

### Brief Historical View of Mechanoregulation in Embryogenesis

Almost a century ago, the concept that mechanical forces regulate embryonic development was gaining momentum. For example, while it had been previously widely believed that smooth muscle cells self-differentiated, by the 1920s the idea that tensional stress-induced elongation of mesenchymal cells was an important stimulus for smooth muscle differentiation in tissues such as the oesophagus was picking up speed (Carey, 1922b). Indeed, Carey et al. suggested that the spiral growth pattern of the epithelium as it expands to form the oesophageal lumen exposed the mesenchyme to extrinsic force which promoted mesenchymal cell elongation and stimulated smooth muscle differentiation (Carey, 1922a). It was later reported that mechanical stress may be important for blood vessel formation and subsequent nutrient supply in developing tissues (Loeschke and Weinhold, 1922; Glucksmann, 1942).

A putative role for mechanically driven processes in embryogenesis was further supported by early observations that embryonic limb explants cultured *in vitro* developed into identifiable bones and joints, but were often incomplete. Indeed, cultured long bones formed a recognisable morphology, but failed to develop a marrow cavity *ex vivo* (Fell, 1925; Thorogood, 1983). These observations led several researchers to suggest that mechanical cues *in vivo* were important in controlling such developmental processes and raised questions about the role of the environment adjacent to the developing bone during morphogenesis (Fell, 1925; Murray, 1926; Drachman and Sokoloff, 1966). Such questions were explored as early as the 1920s when the developing pig femur was used to show preferential osteoblast differentiation in regions under tensile stress (Carey, 1922a). Indeed, during limb rotation, muscular activity causes bending of the femur. As a result, bone is first deposited on the convex aspect of the femoral shaft, which is under high tensile stress, whilst osteoblast differentiation on the concave aspect is secondary. These findings were later confirmed in the chick in the early 1940s when Glucksmann et al., showed that osteogenesis is promoted by tension in chick bone rudiments cultured *in vitro.* Here, the authors cultured chick tibiae rudiments, which naturally became enclosed in a fibrous capsule during the culture period. The fibrous capsule contracted during cultivation and pulled the rudiments together, which altered forces in the capsule, and drove new bone deposition in the direction of increased tension forces (Glucksmann, 1942). Later landmark studies in the 1990s investigated the effect of pharmacologically paralysing both avian and murine embryos at various stages (Hall and Herring, 1990; Rodríguez et al., 1992). Immobilised embryos were shown to have smaller and lighter skeletal bones and less surrounding muscle compared to untreated controls (Hall and Herring, 1990; Rodríguez et al., 1992). Notably, areas with the greatest reduction in musculature, such as around the clavicle, were correlated with a more significant reduction in bone growth, suggesting an integral role for muscle contraction in bone development (Hall and Herring, 1990). Collectively these studies demonstrate that mechanical load is important for both bone development and achieving proper tissue size (Pai, 1965; Hall and Herring, 1990; Rodríguez et al., 1992).

Early studies of cartilage and joint development also used paralysis models to gain insights into the role of mechanical cues in their formation (Fell and Canti, 1934; Murray and Smiles, 1965; Drachman and Sokoloff, 1966; Hall, 1979). For instance, secondary cartilage was found not to form in the quadratojugal bone of immobilised 10-day old chick embryos *in ovo* (Hall, 1979). The authors concluded that continued differentiation of the progenitor pool into chondroblasts as opposed to osteoblasts requires biomechanical signals in the form of muscle contraction. In this case, a lack of movement reduced the mitotic activity of periosteal progenitor cells, which depleted the available pool of progenitor cells with the potential to undergo chondrogenesis (Hall, 1979). This was supported by further studies in mammals which similarly showed that although cartilage formation in the mandible could be initiated in the absence of normal *in vivo* mechanical cues, the maintenance of secondary cartilage required mechanical stimulation. Indeed, in the absence of mechanical stimulation cartilage in *ex vivo* cultured mandibles disappeared as the progenitor cells switched to osteogenesis (Fang and Hall, 1997). Evidence from the 1960s also showed that muscle contraction was indispensable for joint cavity formation; *in ovo* treatment of chick embryos with neuromuscular blocking agents, or complete removal of the lumbosacral spinal cord resulted in absent knee and ankle joint cavities (Drachman and Sokoloff, 1966). Instead, the interzone between articular elements was filled with vascular connective tissue, which eventually became compact and fibrous.

### Intrinsic Forces in Embryonic Development

In addition to extrinsic forces, intrinsic forces such as cell density, shape and ECM compliance also control morphogenesis and cell differentiation within the embryo (McBride et al., 2008; Chevalier et al., 2016a). For instance, mesenchymal condensations are necessary for the development of muscle, bone, cartilage, lung, hair follicles and kidney, and can affect both the physical and biochemical cellular environment (Dunlop and Hall, 1995; McBride et al., 2008). These two elements are often intertwined; for example, an increase in cell density and subsequent round morphology promotes cell-cell adhesion and increases paracrine signalling (Dunlop and Hall, 1995; Knothe Tate et al., 2008). Importantly, each cell within the condensing mesenchyme is likely to encounter a unique set of biophysical cues, as mesenchymal cells are not a homogenous population due to the asymmetry of condensation boundaries (Knothe Tate et al., 2008).

Moreover, just as cell shape and density are important for driving MSC fate decisions, cells receive similar cues during condensation events in embryogenesis, which also act as important regulators of fate. Condensation can occur as an “aggregation” event, in which mesenchymal cells become compressed around a central point, or as an “expansion” event in which a central mitotic mesenchymal pool increases the cell number within a given space (Knothe Tate et al., 2008). During osteogenesis, condensation events are key for increasing the number of pre-osteoblasts, which then differentiate and deposit bone matrix (Dunlop and Hall, 1995). Mesenchymal condensation is also critical for odontogenesis (Mammoto et al., 2011). During the bud stage of tooth formation, neural crest cell (NCC)-derived mesenchyme rapidly proliferates, creating a compact mass of cells with a round morphology (Mammoto et al., 2011). Culture of primary murine NCC isolated from the first pharyngeal arch on micro-patterned substrates revealed that a round cell shape is sufficient to upregulate the odontogenic marker paired box 9 (*Pax9*) independently of cell-cell contact. Specifically, the rounded cell shape suppressed RhoA and cytoskeletal pre-stress within the cell, promoting Pax9-mediated osteogenesis (Mammoto et al., 2011).

Intrinsic biophysical cues also impact on processes such as neurulation. The genetic basis of neural tube closure is relatively well-characterised and to date over 300 genes have been implicated, including Shh, GLI family zinc finger 3 (Gli3), VANGL planar cell polarity protein 2 (Vangl2), zic family member 2 (Zic2), and LDL receptor related protein 2 (Lrp2) (Copp and Greene, 2013; Kur et al., 2014; Galea et al., 2018, 2017; Juriloff and Harris, 2018). Mutations in these genes can predispose sufferers to a range of neural tube defects, such as anencephaly and spina bifida (Galea et al., 2017; Nikolopoulou et al., 2017). In addition to these genetic factors, mechanical cues are required to convert the flat ectoderm into a round tube (Vijayraghavan and Davidson, 2017). Two mechanical processes particularly important for effective neurogenesis are convergent extension (CE) and apical constriction (AC) (Inoue et al., 2016; Nikolopoulou et al., 2017). The neural plate is shaped via CE, which increases the embryonic length in the anterior-posterior direction relative to its medio-lateral width (Vijayraghavan and Davidson, 2017). In late gastrula stage *Xenopus*, the stiffness of dorsal neural tube explants increases from 13 to 85 Pa during CE (Zhou et al., 2009). Treatment of the dorsal neural tube isolates with a ROCK inhibitor resulted in a 50% reduction in tissue stiffness (Zhou et al., 2009). This suggests that cytoskeletal tension accounts for some, but not all, of the stiffness increase that occurs during CE. The same group later generated force-maps of the dorsal explants during CE by using a gel force sensor system, in which explants were embedded into agarose gels containing fluorescent beads. The forces produced by the explant as it underwent CE were inferred by measuring the bead displacement and degree of agarose gel deformation (Zhou et al., 2015). The greatest agarose deformation was observed at the anterior and posterior regions of the dorsal explants. Furthermore, when explants were cultured in stiffer agarose gels, the stress produced by the dorsal explant itself also increased. Thus, the dorsal neural tube is able to respond to and counterbalance changes to its surrounding mechanical environment (Zhou et al., 2015).

Apical constriction (AC) events are critical for driving processes such as gastrulation, neural tube closure, the formation of the salivary glands and inner ear (Sawyer et al., 2010; Inoue et al., 2016; Hartl et al., 2019). During AC, the apical side of the cell contracts, creating cells with a wedge-like morphology. In addition, actin and NMM II accumulate at the cell apex and at cell-cell junctions (Sai and Ladher, 2008; Galea et al., 2017; Vijayraghavan and Davidson, 2017; Butler et al., 2019). Numerous studies in *Xenopus* and vertebrates state that AC and actomyosin contractility are required to regulate the bending and folding of the neural plate and formation of the medial hingepoint (Zhou et al., 2009; Inoue et al., 2016; Nikolopoulou et al., 2017; Suzuki et al., 2017; Butler et al., 2019; Karpińska et al., 2020). The posterior neuropore (PNP) at the most caudal end of the neural tube is known to be under tension during closure. Galea et al. (2017) identified the presence of a F-actin cable around the borders of the neural folds. Laser ablation of the PNP zippering point caused the neuropore to widen and the neural folds to move further apart (Galea et al., 2017). The same group later reported that *ex vivo* ROCK inhibition of E9.5 embryos slows PNP closure by reducing the accumulation of apical F-actin in the neuroepithelium and along the neural folds (Butler et al., 2019). Laser ablation of F-actin cables at the PNP zippering point confirmed that lateral tissue recoil in ROCK-inhibited embryos was greatly reduced compared to controls, therefore ROCK inhibition decreases the tensions that normally act on the neural folds (Butler et al., 2019). In addition, the absence of ROCK activity prevented AC, as quantified by an increase in the apical size of neuroepithelial cells in Y-27632-treated embryos (Butler et al., 2019).

Both contraction of the apical cell surface via actomyosin interactions and removal of the surface membrane are required for effective AC. Several studies have identified proteins such as vinculin and MARCKS that mediate the actomyosin contractility during neural tube closure (Morriss-Kay and Tuckett, 1985; Stumpo et al., 1995; Xu et al., 1998). For instance, the protein Catulin A is a key player in Rho-mediated AC; *Catulin A-/-* mutants are embryonically lethal at E10.5 and neural tube fusion fails to occur at the hindbrain/cervical boundary (Karpińska et al., 2020). Apical actin and nestin filaments did not form in the neuroepithelium of mutants, which was correlated with a lack of active RhoA signalling (Karpińska et al., 2020). A recent study identified the endocytic receptor Lrp2 as an integral mediator of membrane remodelling during AC. Indeed, a striking increase in apical surface area, defective neural fold morphogenesis and mis-localisation of the planar cell polarity protein Vangl2 were all observed in Lrp2 mutants (Kowalczyk et al., 2021).

### Measuring Embryonic Stiffnesses *in vivo*

*In vivo*, ECM and cellular stiffness can affect cell fate decisions and techniques for measuring these nano- and micro-scale tissue elasticities are advancing. However, measuring mechanical properties *in vivo* is very challenging and few studies have directly quantified stiffness within the embryo (Barriga et al., 2018; Wozniak and Chen, 2009; Efremov et al., 2011; Marturano et al., 2013; Chevalier et al., 2016b). The Young’s modulus of a tissue can be indirectly estimated via micropipette aspiration assays, in which several cells within a tissue are sucked into a micropipette. Using this approach, the length of the aspirated tissue at a given suction pressure can be used to infer cellular mechanical properties (Majkut et al., 2013; Daza et al., 2019). For example, Majkut et al. (2013), demonstrated that *in vivo*, mouse heart tissue stiffens over time, which is important for contraction of cardiomyocytes. The same study showed that *in vitro* culture of primary cardiomyocytes from E4 embryos on collagen I substrates that closely resemble the stiffness of the heart at this stage (1–2 kPa) initiated their contraction.

Measurement of cell and ECM stiffness *in vivo* is also possible via AFM force spectroscopy (Thurner, 2009; Iwashita et al., 2014; Chevalier et al., 2016b; Koser et al., 2016). AFM measures the deflection of a laser beam focussed on the back of a cantilever as it indents the surface of a tissue (Alonso and Goldmann, 2003). The deflections are captured by a photodiode and used to infer stiffness. A hallmark study in chick identified that the stiffness of the embryonic tendon significantly increased over time at both the nano- and micro-scale between stage HH38 and HH43. Inhibition of enzymatic collagen cross-linking identified that this decrease correlated with an increase in collagen cross-linking and was necessary for tendon development (Marturano et al., 2013). In the same year, Iwashita and colleagues reported a correlation between matrix stiffness and cell fate in the murine cortical brain. From E12.5 to E18.5, the stiffness of each cortical brain layer significantly increased as neuronal differentiation progressed. This shift in stiffness was attributed to both cellular and matrix origins, as *in vitro* AFM measurements confirmed neuronal and matrix stiffness changes independently (Iwashita et al., 2014).

## Summary and Outlook

In summary, mechanical cues play a fundamental role in driving both adult and embryonic cell fate decisions. Despite significant progress in understanding the molecular mechanisms that govern mechanotransduction, many of the signalling pathways remain to be defined. Extrinsic cues such as fluid flow and compression as well as local intrinsic cues such as cell shape and density are “felt” by mechano-sensors at the cellular-ECM interface. This activates various downstream signalling pathways including Rho/ROCK signalling, which promotes actomyosin rearrangements and allows cells to counteract the forces from their surrounding microenvironment. Although the cytoskeleton is known to play an integral role in translating cues from the ECM to the cell and *vice versa*, the intricacies of nuclear mechanotransduction are only now becoming apparent. Moreover, the discovery that the nucleus can deform independently of the cytoskeleton in response to directly applied forces, thereby increasing nuclear entry of factors such as YAP, demonstrates the far-reaching effects of mechanical stimuli (Elosegui-Artola et al., 2017).

Understanding the impact of intrinsic mechanical cues, such as stiffness, during embryogenesis is somewhat limited by the tools available to study very soft tissues. Indeed, for embryonic tissues, experimental measurements of stiffness can be limited by challenges regarding sample preparation and immobilisation, as slicing can often disrupt tissue integrity and structure (Viji Babu and Radmacher, 2019). In addition, embryonic samples are composed of highly heterogeneous morphological structures, which can hinder accurate measurements of tissue stiffness using techniques such as AFM (Galluzzi et al., 2018). Despite this, several recent reports describe how AFM can be optimised to measure the stiffness of soft culture surfaces and tissues (Galluzzi et al., 2018; Babu et al., 2019; Norman et al., 2021).

With the development of new techniques such as the standardised nanomechanical AFM procedure, which standardises AFM calibration and protocols between laboratories, reproducible data acquisition, particularly on soft tissue samples should become the norm (Schillers et al., 2017). Recent advancements also include magnetic devices capable of measuring the viscoelastic properties of entire 3D structures up to the size of an E10.5 mouse embryo (Zhu et al., 2020). The device generates a magnetic field to displace magnetic beads injected into the developing mouse limb bud, and has been used to uncover the presence of a mesodermal stiffness gradient (Zhu et al., 2020). In addition to improving our understanding of mechanical cues in embryonic development, measuring tissue stiffness is likely to play an increasingly important role in non-invasive diagnosis of cancer (including extent of invasion), liver fibrosis and primary biliary cholangitis (Corpechot et al., 2021; Li and Wu, 2021; Shao et al., 2021). For instance, shear wave elastography ultrasound imaging can detect increases in the Young’s modulus of tissues induced by malignant tumours and is being optimised for use clinically through the addition of colour mapping functionality (Lee et al., 2020).

In recent years, there have also been growing efforts to re-create mechanical cues experienced by living tissues in 3D engineered tissue constructs grown *in vitro*. However, whilst specific elasticities can often be engineered into polymer scaffolds, matching the mechanical cues experienced by cells within native tissues is often more challenging. This is because many tissue do not behave elastically, but rather display time-dependent and non-linear responses (Chaudhuri et al., 2020; Elosegui-Artola, 2021). For example, rather than immediately returning to their original shape when an applied strain is removed, tissues are viscoelastic and exhibit a time-dependent response (Chaudhuri et al., 2020; Efremov et al., 2020; Pogoda et al., 2021). To create materials that better reflect these tissue responses, hydrogels with dynamic cross-links between polymers have been developed (Chaudhuri et al., 2020). For example, within these materials, covalent thioester exchange and/or hydrozone bonds allow for investigation of time-dependent rearrangements of bonds (Brown et al., 2018; Marozas et al., 2019).

Finally, gaining a better understanding of the cellular response to local matrix compliance and topography has important implications for improving *in vitro* differentiation assays. This will in turn improve the design of physiologically relevant materials for tissue repair. This has particular relevance in orthopaedic applications, such as knee arthroplasty for osteoarthritis patients. Here, appropriate implant structure and mechanical stimulation may be necessary to promote its anchorage within the bone (Li et al., 2018). The upcoming challenge in tissue engineering will be not only to understand the complexity of the cellular response to mechanical cues, but also to develop scaffolds that accurately capture and recapitulate *in vivo* environments.

## Author Contributions

Both authors listed have made a substantial, direct and intellectual contribution to the work, and approved it for publication.

## Conflict of Interest

The authors declare that the research was conducted in the absence of any commercial or financial relationships that could be construed as a potential conflict of interest.

## Publisher’s Note

All claims expressed in this article are solely those of the authors and do not necessarily represent those of their affiliated organizations, or those of the publisher, the editors and the reviewers. Any product that may be evaluated in this article, or claim that may be made by its manufacturer, is not guaranteed or endorsed by the publisher.
